# Case Report: Acute Renal and Splenic Infarctions Secondary to Atrial Fibrillation

**DOI:** 10.3389/fcvm.2022.879322

**Published:** 2022-05-24

**Authors:** Li Yihan, Fan Guanqi, Hu Tong, Ge Junye, Jingquan Zhong, Tongshuai Chen

**Affiliations:** Key Laboratory of Cardiovascular Remodeling and Function Research, Chinese Ministry of Education, Chinese National Health Commission and Chinese Academy of Medical Sciences, State and Shandong Province Joint Key Laboratory of Translational Cardiovascular Medicine, Department of Cardiology, Qilu Hospital, Cheeloo College of Medicine, Shandong University, Jinan, China

**Keywords:** renal infarctions, splenic infarctions, atrial fibrillation, Radiofrequency ablation, left atrial appendage occlusion

## Abstract

Acute renal and splenic infarctions are an uncommon condition that can result from obstruction or decrease of renal and splenic arterial flow. We described a 73-year-old woman who presented with right flank pain and nocturnal dyspnea. The computed tomography (CT) scan with intravenous contrast showed multiple infarcts in both bilateral kidneys and spleen. Serum creatinine clearance was impaired. Further investigation by electrocardiogram (ECG) and 24-h Holter revealed that the patient had paroxysmal atrial fibrillation (PAF). Transthoracic and transesophageal echocardiographic findings were unremarkable except for severe spontaneous echo contrast (SEC) in the left atrial appendage. The development of thromboembolic renal and splenic infarction was attributed to embolism caused by atrial fibrillation. Anticoagulant therapy was initiated with low molecular weight heparin (LMWH) and followed by an oral anticoagulant. To manage PAF and prevent further embolism, the “One-stop” procedure, including atrial fibrillation catheter ablation and left atrial appendage occlusion (LAAO), was applied to this patient. Follow-up at 1 month showed normal sinus rhythm, improved renal function, and relieved renal and splenic infarction.

## Introduction

Acute renal infarctions (RI) and splenic infarctions (SI) are rare events; the incidence of which is estimated to be 0.004–0.01% in hospitalized patients (1), and delayed diagnosis or misdiagnosis often leads to rapid deterioration of the patient (2). The initial symptoms of both RI and SI often begin with a sudden onset of severe flank pain. Patients may also present with non-specific symptoms, including low-grade fever, nausea, vomiting, and frequent elevation of lactate dehydrogenase (LDH) levels (3). Enhanced CT imaging is the gold standard for the diagnosis of RI and SI (4). It is generally agreed upon that the causes of acute renal infarction are renal artery lesions, atrial fibrillation (AF), valvular or ischemic heart disease, peripheral vascular disease, and hypercoagulable state, among which AF seems to be the leading cause, but its frequency differs among studies (5, 6). AF is also the most common associated predisposing condition in SI (7). Virchow's triad at the AF state produces imbalances between thrombotic and thrombolytic factors, leading to the formation of embolus, which may dislodge and travel through the systemic circulation, finally resulting in embolism in the end organs (8). If the diagnosis of acute RI is missed initially or delayed, patients are at risk of renal failure, which can lead to death (9).

We present a patient with embolic renal and splenic infarction secondary to atrial fibrillation. Anticoagulation therapy was given according to the CHA2DS2VASc score, and then, a “One-stop” procedure, including atrial fibrillation catheter ablation and left atrial appendage occlusion, was applied to restore sinus rhythm and prevent further embolism.

## Case Presentation

A 73-year-old female patient (height:153 cm, weigh:45 kg) was presented to our emergency department with the new symptom of right flank pain for 10 days, accompanied by palpitation and nocturnal dyspnea, the patient suffered a cerebral infarction 25 years ago, but no medication has been taken routinely. Before admission, the patient received drug therapy, including diuretic, antiplatelet, hepatic protectant, etc., for diagnosis of atrial fibrillation and heart failure in a county hospital, but failed to improve symptoms.

**Figure 1:**
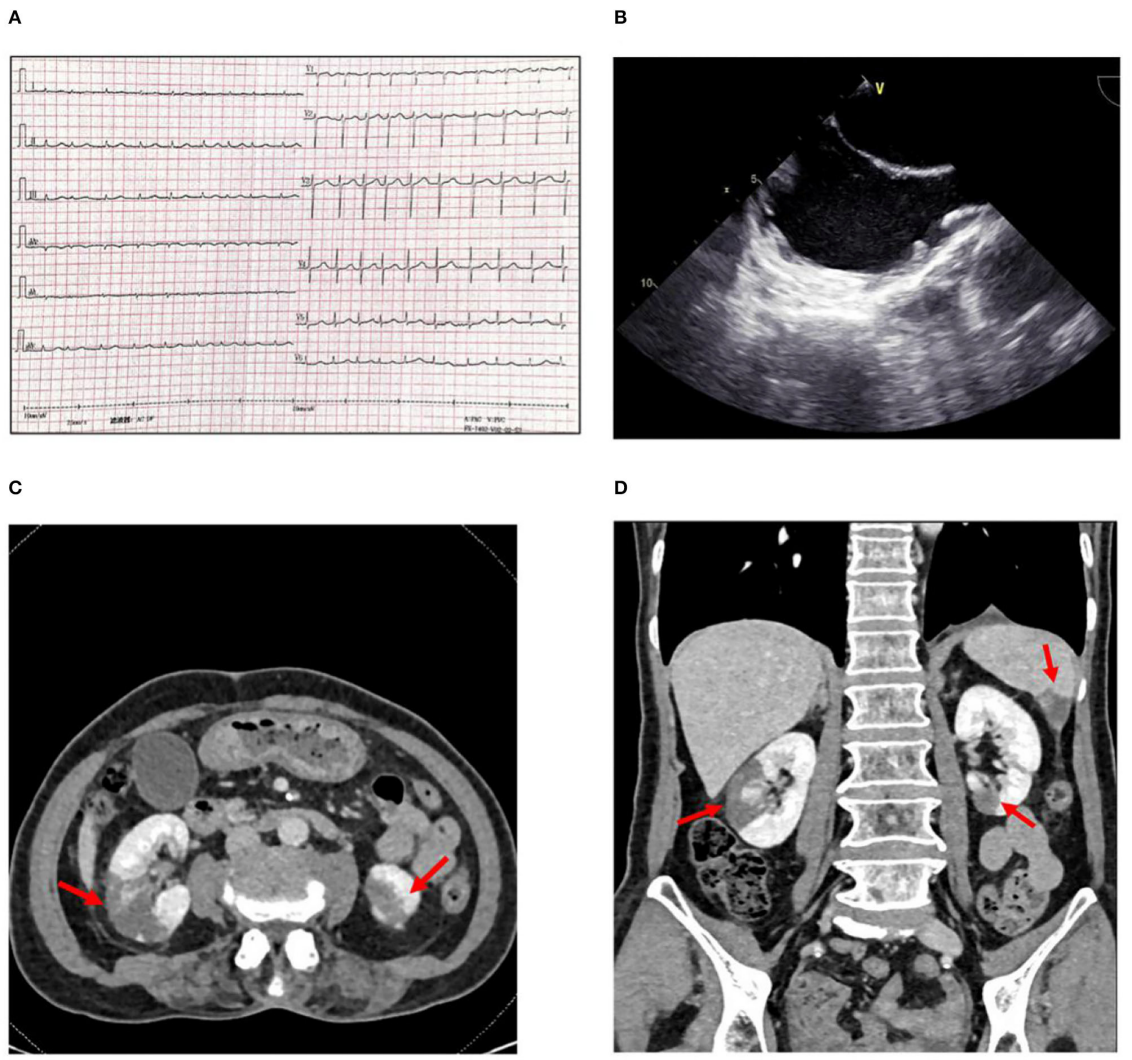
**(A)** Electrocardiogram showing atrial fibrillation with a rapid rate. **(B)** Transesophageal echocardiography showing spontaneous echo contrast (SEC) in left atrium and atrial appendage. **(C)** Abdominal CT scan with intravenous contrast shows infarctions (arrows) in the middle right and **(D)** Inferior left renal parenchyma and the inferior aspect of the spleen.

Her vital signs were all normal except for the HR was 114 bpm. A physical examination revealed that the heart rhythm was irregular, and the ECG showed atrial fibrillation (AF) with a rapid ventricular rate(Figure 1A). Her white blood cell count was 15.55 × 10^9^ per L, red blood cell was 3.87^*^10^∧^9/L, and platelet count was 171^*^10^∧^9/L, respectively. A urinalysis showed negative protein, 23.5 white blood cells (<25/ul), and 24.5 red blood cells (<25/ul), within normal limits. LDL-C was 3.91 mmol/L. The D-Dimer was over 20 ug/ml (<0.5 ug/ml). The blood creatinine concentration was 116 umol/L, and GFR calculated by Cockcroft-Gault was 27.27 ml/min. The liver function tests were abnormal: Serum alanine aminotransaminase (ALT) was 808 IU/L (0–40 U/L) and aspartate aminotransaminase (AST) was 1112 IU/L (0–40 U/L). Also, the total bilirubin, indirect bilirubin, direct bilirubin, and albumin levels were normal (19 umol/L, 10 umol/L, 0 umol/L, and 39 g/L, respectively). The N-terminal b-type natriuretic peptide precursor (NT-proBNP) was 4,577 pg/ml (normal range, ≤ 900 pg/ml aged 50–75).

With consideration of her complaint about abdominal discomfort, two possible causes include (1) hepatalgia caused by congestion of the liver, and (2) high possibility of detachment of thrombosis during atrial fibrillation cardioversion, which can cause mesenteric artery embolism and intestinal ischemia. A mesenteric artery CT angiography (CTA) was done, mesenteric artery CTA showed a mild to moderate stenosis in the trunk section due to eccentric thickening of the vascular wall, with focal calcification. Additionally, multiple infarcts were detected in both kidneys and spleen, but no obvious thrombosis in the renal artery and splenic artery (Figures 1C,D). Transthoracic echocardiography (TTE) revealed all four chambers of the heart were of normal sizes with a left atrium dimension of 2.8 cm. Transesophageal echocardiography (TEE) was performed for suspected intracardiac thrombus formation; there was no intracardiac thrombus, but a severe spontaneous echo contrast (SEC) in both atrium and atrial appendage (Figure 1B). Holter monitoring recorded the whole episodes of atrial fibrillation at 24-h follow-up. Interestingly, the patient's CHA2DS2VASc score was six based on her age, sex, hypertension, vascular disease, and thromboembolic events: one point for age 65–74 years, one point for female, one point for a history of hypertension, one point for peripheral artery disease, and two points for the current embolic event. Also, the patient's HAS-BLED score was three based on her age, hypertension, and liver function abnormality; one point for age >65 years, one point for a history of hypertension, and one point for ALT > three times the upper limit of normal.

**Figure 2:**
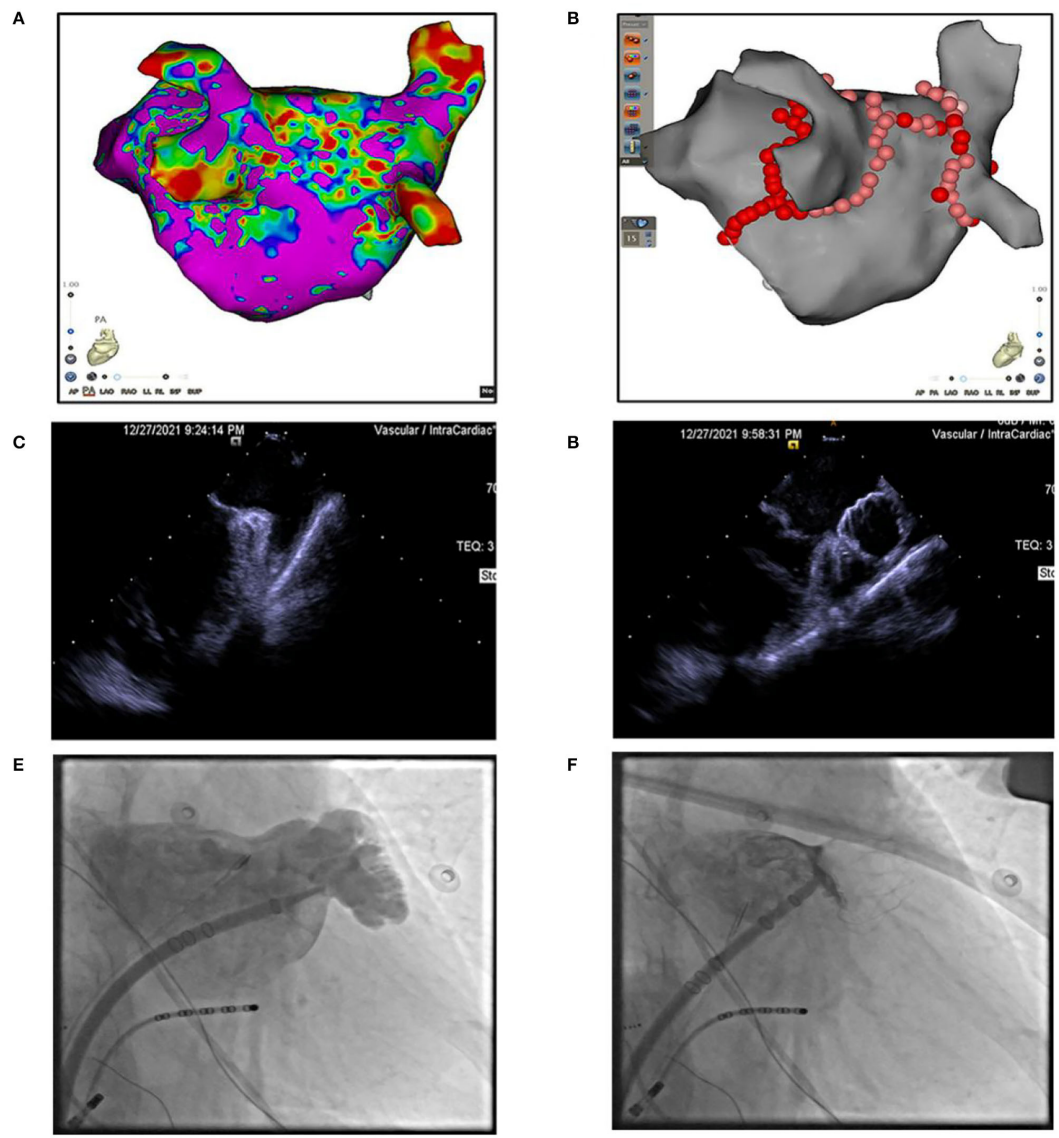
**(A)** 3D modeling and substrate mapping of left atrium. **(B)** Catheter ablation at pulmonary veins, left atrium roof, and mitral isthmus. **(C,D)** Intracardiac echocardiography image of left atrial appendage (LAA) before or after LAA occlusion. **(E,F)** Fluoroscopy image of left atrial appendage (LAA) before or after LAA occlusion.

**Figure 3:**
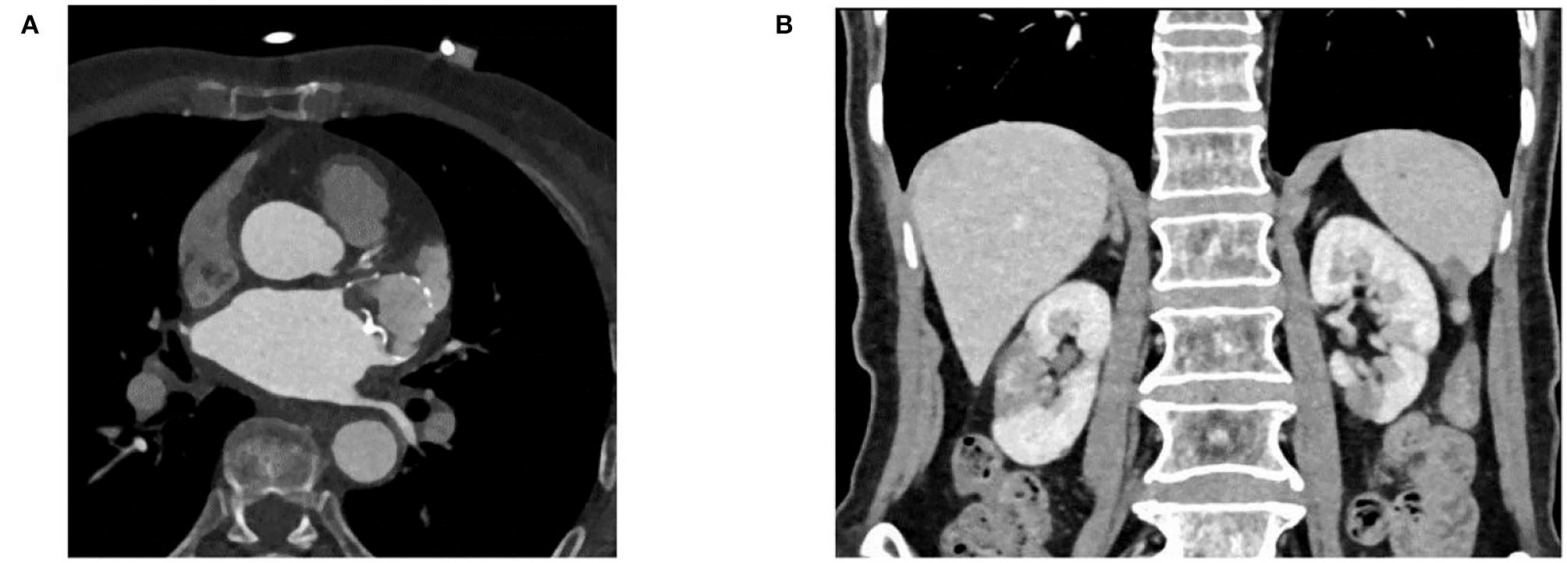
**(A)** Coronary artery CT angiography showing no protruding device-related thrombus and peri-device leak. **(B)** Renal artery CT angiography showing no new onset infarction.

Anticoagulation therapy was initiated with an enoxaparin sodium injection of 120 mg/day, and then, switched to rivaroxaban 20 mg/day. Drug cardioversion of AF including amiodarone intravenous injection failed, and the rapid heart rate was hard to control by β-blockers, digitalis, etc. An electrophysiological study and catheter ablation was recommended to restore sinus rhythm for the AF refractory to drug therapy. Percutaneous left atrial appendage occlusion has also been considered to decrease the risk of stroke in this patient. After a combination of ablation (Figures 2A,B) and left atrial appendage occlusion (LAAO) (Figures 2C-F) as a “One-stop” procedure, the patient restored sinus rhythm. The patient's symptoms quickly improved, and the patient was discharged 2 days later. After 1 month of follow-up, the patient complained of no special discomfort, and the holter showed sinus rhythm. GFR was 35.23 ml/min (27.27 ml/min when first examined). The liver function improved: ALT was 26 IU/L (0–40 U/L) and AST was 42 IU/L (0–40 U/L). Coronary artery CT angiography showed no protruding thrombi around the occlusion device and peri-device leak (Figure 3A). Renal artery CT angiography showed no new-onset infarction, the range of old infarcted lesions got smaller, and the margin of renal and splenic infarction got irregular (Figure 3B). The patient continued oral anticoagulation of rivaroxaban for 45 days after discharge, followed by 6 months of dual platelet inhibition, with clopidogrel 75 mg and lifelong continuation of ASA 100 mg/day alone. We will follow up with the patient in 1 month, 3 months, and 6 months after surgery. Then, she will be followed up every 6 months.

## Discussion

Renal infarction and splenic infarction are rare events in clinical practice. Patients with renal infarction and splenic infarction are often confused with urinary tract infection (UTI), nephrolithiasis, biliary pathologies, appendicitis, and mesenteric ischemia because they do not have specific clinical symptoms. Clinicians should have a low threshold for suspicion of RI and SI because of the difference in the way it is treated compared to other diagnoses for these non-specific symptoms.

Renal infarction can affect one or two kidneys to varying degrees. In a retrospective study by Yang et al., 19.1% of the patients had bilateral renal infarction. Moreover, they reported that 34.8% of the patients with acute renal infarction had a renal failure at the time of diagnosis and 27.4% of patients with renal infarction are at risk for developing CKD (10). Nagasawa et al. reported that the size of the renal artery orifice may be a factor that contributes to the laterality of ARI, and assessment of anatomical features is important when considering the laterality of the disease (11). Also, the volume of the infarction may be a factor in the degree of renal function decline in ARI, and assessment of infarct volume in ARI is important (12). It was noted that acute kidney injury(AKI)is often associated with acute renal infarction. Although most AKIs recover spontaneously, renal impairment following acute renal infarction can persist. Thus, early diagnosis and intervention are needed to preserve renal function. Splenic infarction is a thromboembolic disease that is also frequently missed in acute settings. Splenic infarction patients often presented with left upper abdominal pain and tachycardia. A history of hypertension, atrial fibrillation, and a laboratory result of leukocytosis or thrombocytopenia may provide a clue for clinicians to include splenic infarction in the differential list. In a retrospective analysis of SI patients (13), SI could be the initial presentation of previously unknown medical conditions in 38% of patients. The main underlying mechanisms were cardioembolic (54.4%), vascular (20%), hematologic disorders (15.6%), and multiple causes (21.1%). Atrial fibrillation and atherosclerosis were common in older patients (age>70 years), while antiphospholipid syndrome occurred exclusively in younger individuals. In addition, pancreatic disorders also appear to be an important cause of splenic infarction, presumably due to the proximity of the pancreas to the splenic vessels (14).

The origin of renal infarction is usually cardioembolic, especially due to atrial fibrillation (15). Atrial fibrillation is present in only 18–64% of patients according to different series, however, it is noted that in 30–60% of patients with renal infarction, no provoking factor is identified (16, 17). A retrospective study reported an additional 10% of the patients were identified with atrial fibrillation during follow-up, suggesting that patients with RI with paroxysmal atrial fibrillation may be underrecognized by routine monitoring. Interestingly, the patients with provoked factors, such as atrial fibrillation had a higher rate of recurrence of arterial thrombosis, during follow-up (18). In a randomized clinical trial, among older community-dwelling individuals with hypertension, AF screening with a wearable cECG monitor was increased AF detection 10-fold and prompted the initiation of anticoagulant therapy in most cases. Compared with continuous ECG, intermittent oscillometric screening with a BP monitor was an inferior strategy for detecting paroxysmal AF (19). So, it is reasonable to apply intensive ECG monitoring for patients with a high risk of thromboses, such as stroke or renal infarction.

Treatment of RI and SI depends on the etiology. To date, there is no guideline regarding the antithrombotic treatment of RI either in the early phase or in long term. Anti-coagulation is a necessary and classical therapy for artery infarction and should be adopted as soon as possible to restore the renal perfusion and improve the prognosis (20). In retrospective studies, patients with RI have an all-cause mortality rate of 19.7% at 40 months, despite a rare evolution to dialysis or end-stage renal disease (2% at 41 months) (21). This means that the poor long-term outcome is not due to the severity of the kidney injury, but mainly to cardiovascular events and patient's comorbidities with an outcome rate of 12% at 48 months (22). Similarly, strokes related to AF are associated with higher mortality and morbidity when compared with non-AF strokes, emphasizing the need for more effective stroke prevention in these patients (23). It has been shown that in patients with non-valvular AF, 90% of thrombi are located in the LAA (24). Left atrial appendage occlusion as means to prevent thromboembolism in AF is based on the observation that the majority of thrombi in non-valvular AF form in this *cul de sac* structure. Left atrial appendage occlusion using interventional techniques has been demonstrated to be equivalent to oral VKAs in reducing thromboembolic events, while reducing the OAC inherent risk for major bleeds. The 5-year outcomes of the PREVAIL trial, combined with the 5-year outcomes of the PROTECT AF trial, demonstrate that LAAO with the Watchman device provides stroke prevention in nonvalvular atrial fibrillation comparable to warfarin, with additional reductions in major bleeding, particularly hemorrhagic stroke, and mortality (25). Therefore, in patients with an elevated bleeding and thrombosis risk who undergo left AF ablation, combining ablation and LAA occluder implantation in one procedure, may be a “reasonable opportunity”: AF ablation requires a transseptal approach, which allows additional occluder implantation using the same left atrial access and, therefore, avoids an additional procedure with the transseptal procedure (26).

In conclusion, acute renal infarction and splenic infarction are rare but very important clinical problems. It must be kept in mind in the differential diagnosis of flank pain and AKI. Patients should be examined in detail in terms of etiological factors and treated properly.

## Data Availability Statement

The original contributions presented in the study are included in the article/supplementary materials, further inquiries can be directed to the corresponding author/s.

## Ethics Statement

The studies involving human participants were reviewed and approved by Medical Ethics Committee of Qilu Hospital of Shandong University. The patients/participants provided their written informed consent to participate in this study. Written informed consent was obtained from the individual(s) for the publication of any potentially identifiable images or data included in this article.

## Author Contributions

LY, FG, TC, and JZ: conceptualization. LY: data interpretation, drafting article. LY, FG, HT, and GJ: critical revision of article. LY, FG, HT, GJ, TC, and JZ: approval of article. All authors contributed to the article and approved the submitted version.

## Conflict of Interest

The authors declare that the research was conducted in the absence of any commercial or financial relationships that could be construed as a potential conflict of interest.

## Publisher's Note

All claims expressed in this article are solely those of the authors and do not necessarily represent those of their affiliated organizations, or those of the publisher, the editors and the reviewers. Any product that may be evaluated in this article, or claim that may be made by its manufacturer, is not guaranteed or endorsed by the publisher.
